# Four-color multiplex real-time PCR assay prototype targeting azithromycin resistance mutations in *Mycoplasma genitalium*

**DOI:** 10.1186/s12879-019-4424-2

**Published:** 2019-09-23

**Authors:** Olivier Thellin, Benaïssa Elmoualij, Willy Zorzi, Jorgen S. Jensen, Renaud Close, Valerie Deregowski, Muriel Le Guern Fellous, Pascale Quatresooz

**Affiliations:** 1 Department of Human Histology-CRPP, University of Liège, Avenue Hippocrate 15, Sart Tilman, 4000 Liège, Belgium; 2 Statens Serum Institut, Artillerivej 5, 2300 Copenhagen S, Denmark; 3 Diagenode s.a., Rue du Bois Saint-Jean 3, 4102 Liège, Belgium

**Keywords:** Real-time PCR, *Mycoplasma genitalium*, Azithromycin resistance, Diagnostic

## Abstract

**Background:**

The worldwide expansion of macrolide-resistant *Mycoplasma genitalium* (MG) in cases of genital infections has led to an increased recurrence rate of these infections after first-line azithromycin treatment. By detecting the presence of azithromycin-resistant MG, the patient’s antibiotic treatment can be targeted and the spread of resistance prevented. With this aim in mind, macrolide-resistance detection kits are helpful tools for the physician.

**Methods:**

Azithromycin resistance mutations in MG are targeted using a four-color multiplex real-time RT-PCR assay. Tested targets include plasmid DNA (as positive controls) as well as macrolide-sensitive and macrolide-resistant genomic DNA from characterized cell lines and clinical samples.

**Results:**

The analytical data presented here were generated from plasmid DNA and genomic RNA/DNA and include adaptation to an internal control, specificity between targets, specificity vs non-MG species, limit of detection (LoD) and interference studies (co-infection and endogenous substances). The clinical data were based on the application of the assay to clinical samples characterized by sequencing.

**Conclusions:**

A new NAAT (nucleic acid amplification test) prototype has been developed in collaboration with the Diagenode s.a. company, this prototype targets MG and azithromycin-resistance mutations in that pathogen.

## Background

*Mycoplasma genitalium* (MG) is responsible for non-chlamydial non-gonococcal urethritis (NCNGU) in both men and women. It is also involved in cervicitis, endometritis and pelvic inflammatory disease (reviewed in [1]). MG is present in only 1 to 3.3% of the non-symptomatic population [1] but its prevalence is higher in patients attending sexually transmitted infection (STI) clinics [2–4] or female sex workers [5]. Due to the absence of a cell wall, MG has few antibiotic targets. Recommended first- and second-line antibiotic treatments are azithromycin (AZM) and moxifloxacin respectively [1]. Treatment with doxycycline may be attempted before treatment with pristinamycin, which stands as the last evaluated active therapy [1, 6]. Azithromycin is usually administered either as a 1 g single dose or as 500 mg on day one followed by 250 mg on days 2 to 5. Both regimens are likely to induce azithromycin resistance in MG [7, 8] as illustrated by a 100% azithromycin-resistant MG population in Greenland, where azithromycin has been widely used [9]. As emphasized by Wise [10], testing of MG and of azithromycin resistance in MG is critical in order for the clinician to select targeted antibiotic therapy [11]. Considering the difficulty of performing MG culture [12, 13], multiple in-house assays or commercial nucleic acid amplification tests (NAAT) have been developed to detect the presence of MG with or without macrolide resistance testing. Resistance to azithromycin is mediated by macrolide-resistance-mediating mutations (MRMM) of the 23S rRNA gene in positions 2058 and 2059 (*Escherichia coli* numbering) [14] and a variety of assays have been described for the detection of these mutations [15–18]. Some NAATs that base MRMM detection on additional Sanger or PyroMark sequencing have also been developed [17, 19]. We here present and test a four-color multiplex real-time PCR assay prototype, targeting total MG with MRMM detection in a homogeneous assay. MRMM targets selected in this assay account for the majority of azithromycin resistance mutations seen in MG clinical samples. This prototype has been developed in collaboration with Diagenode s.a. and has served as a basis for the creation of the commercial S-DiaMGRes in vitro diagnostic kit.

## Methods

### Primers and probes

The primers and probes (P/P) used in this paper were designed in collaboration with the Diagenode s.a. company and have been integrated in the S-DiaMGRes in vitro diagnostic (IVD) kit. Therefore, exact sequences for these primers and probes will not be given here for intellectual property reasons.

P/P for the detection of total MG specifically target the Mg219 gene and have the same sequences as the Mg219 P/P used in the S-DiaMGTV™ IVD kit (Diagenode s.a., Belgium). The probe for this target is coupled to the ROX dye.

P/P for the detection of AZM-resistant MG (mutants) or AZM-sensitive MG (wild type, WT) were designed to target positions 2058 and 2059 (*E. coli* numbering) of the 23S rRNA gene which are known to trigger AZM-resistance. The probes targeting mutations of the 2058 nucleotide (A2058C, A2058G and A2058T) are coupled to the FAM dye. The probes targeting mutations of the 2059 nucleotide (A2059C and A2059G) are coupled to the Yakima Yellow (YY) dye. The primers are shared between the WT and each mutant, while the probes provide detection specificity.

In addition, an internal control (IC) for the entire process from the extraction to the RT-PCR (Extraction & Inhibition Control, or EIC: a virus, added to the sample prior to extraction) or for the PCR only (Universal Inhibition Control, or UIC: plasmid DNA including the EIC genomic DNA target sequence, added to the PCR well) is detected using P/P that include a probe coupled to the Cy5 dye. These internal controls are standard complements to PCR IVD kits from Diagenode s.a. and were used according to the manufacturer’s instructions.

### Plasmid and genomic DNA

Plasmid DNA for each target was purchased from GenScript (USA), with each target sequence inserted into a separate pUC57 plasmid.

Genomic DNA from WT and mutant MG were provided by Dr. Jorgen Jensen (Copenhagen, Denmark) from the following cultured strains of MG: M30 (WT), M2300 (WT), M2321 (WT), M2341 (WT), M6302 (A2058C), M6271 (A2058G), M6604 (A2058G), M6926 (A2058T), M6303 (A2059G) and M6320 (A2059G).

Genomic DNA from non-MG species were obtained from ATCC/LGC Standards (France).

### RNA/DNA extractions

Extractions of total RNA/DNA from biological matrices for analytical studies were processed on a MagNA Pure MP96 automatic extractor (Roche, Belgium) according to manufacturer’s instructions, using a MagNA Pure 96 DNA and Viral NA Large Volume 2.0 kit and the Pathogen Universal 500 3.1 protocol.

Extraction of total RNA/DNA from MG strain cultures for analytical studies was performed using Qiagen DNeasy Blood & Tissue Kit (Qiagen, Hilden, Germany).

The primary clinical samples had been stored at − 20 °C for up to 3 years. Swab samples were extracted using the MagNA Pure Universal Pathogen 200 protocol with a MagNA Pure 96 DNA and Viral NA Small Volume Kit (Roche Diagnostics A/S, Hvidovre, Denmark) using 200 μL of the sample. Urine samples were extracted by centrifugation of 1 mL of urine in a sterile 1.5 mL Eppendorf tube at 20000×*g* for 15 min. The pellet was resuspended in 200 μL sterile PBS and all 200 μL were used for extraction, as for the swab samples.

### Real-time PCR and real-time RT-PCR

Real-time PCR (analytical data on plasmid DNA) and real-time RT-PCR (clinical data on bacteria present in clinical samples) were both performed with the following RT-PCR protocol: 10 min at 48 °C (reverse transcription step), 7 min at 95 °C (RT inactivation / DNA initial denaturation) and 50 amplification cycles of denaturation for 10 s at 95 °C, annealing for 15 s at 61 °C and extension for 30 s at 68 °C. RT-PCR reactions were performed using the Path-ID™ Multiplex One-Step RT-PCR Kit master mix (Life Technologies, Belgium) according to manufacturer instructions, with a total reaction volume of 25 μL per well and a sample volume of 5 μL per well. The RT-PCR reactions were performed on ABI 7500 Fast cyclers (ABI Applied Biosystems, Belgium and Denmark).

### Limits of detection

The matrices used to perform limit of detection (LoD) studies were the ESwab Liquid Amies Collection and Transport System (Copan, Belgium) and an anonymized mix of urine samples (Seralab, Belgium). The LoD were calculated by performing probit analyses using Minitab 17 software (Minitab, France). Values at 95% were selected as the LoD.

### Co-infection interferences and endogenous substance interferences

The RT-PCR results of co-infection interference studies and of endogenous substance interference studies are interpreted according to the interference criteria summarized in Table 1.

**Table 1:** Interference criteria interpretation grid for co-infection interference studies

Reference condition	Test condition		Conclusion
% of positive calls	% of positive calls	ΔCt	
< 85%	Any %	Any ΔCt	The experiment is invalid. The causes of the shift in sensitivity must be investigated and the experiment reconducted.
≥ 85%	< 85%	Any ΔCt	The highly-concentrated DNA or the tested substance is considered to be interfering with the detection of the target pathogen if any of these 2 criteria are met.
	Any %	ΔCt ≥ 2SDrep	
≥ 85%	≥ 85%	Any ΔCt	The highly-concentrated DNA or the tested substance is considered not to be interfering with the detection of the target pathogen if both criteria are met.
	Any %	ΔCt < 2SDrep	

% of positive calls: % of wells with target detection; ΔCt: Ct difference between reference condition and test condition; 2SDrep: 2× the standard deviation calculated during the limit of detection study for the target concentration closest to the concentration used here

For co-infection interference studies, the reference condition is the RT-PCR with only the low-concentration target in the PCR well. The test condition is the RT-PCR with both the low-concentration target DNA and the high-concentration interfering DNA in the PCR well.

For the endogenous substance interference studies, the reference condition is the RT-PCR without addition of the tested substance in the matrix. The test condition is the RT-PCR with addition of the tested substance in the matrix.

### Testing of clinical samples

The optimized real-time RT-PCR multiplex assay with internal amplification control was tested on 43 stored anonymized clinical samples received by the Statens Serum Institut (Copenhagen, Denmark) for diagnostic testing for MG*.* The internal control was added to the master mix and not to the primary sample; thus, it was not subject to the DNA extraction procedure. The selected samples aimed to represent both wild-type (WT) macrolide susceptible strains and strains with macrolide resistance mediating mutations. Based on the routine diagnostic test results of a pyrosequencing assay [20], the samples represented mutations as shown in Table 4, and three were MG negative. Quantitative results were produced for both PCR assays based on a standard curve generated from genomic MG DNA.

### Ethical considerations

There is no need for ethical approval of the study itself by an Ethical Review Board as it involves only de-identified samples. As samples were anonymized and were examined here with the same purpose as they were originally submitted, the study is considered quality assurance or quality development and thus, ethics approval is not needed according to Danish law no. 593 of 14. June 2011 with subsequent amendments about ethics approval of research in health sciences.

## Results

### Specificity between targets

A multiplex RT-PCR targeting the 2058 and 2059 nucleotides of the gene coding for the 23S rRNA was performed to check the specificity of the P/P for their targets. The P/P that were included in the multiplex were those targeting the 2058 mutants MG (FAM dye), the 2059 mutants MG (YY dye), the non-mutated WT MG (ROX dye) and the UIC that was added to each PCR well (Cy5 dye). The multiplex was tested against plasmid target DNA and against genomic DNA from MG strains, each at 1250 copies/well. All wells showed a positive signal for the UIC, validating the PCR results. For each tested DNA, a signal was obtained in the expected channel and only in that channel, validating the specificity of the P/P between absence of mutation (WT), mutation of 2058 nucleotide and mutation of 2059 nucleotide (*n* = 3). Please note that no A2059C MG strain was available for testing. Therefore, the P/P for that mutation could only be tested against plasmid DNA.

### Impact of the addition of an internal control

The addition of EIC or UIC in the RT-PCR process may affect the signal obtained for each of the targets. To evaluate this impact, we compared 2 multiplexes. Both multiplexes included P/P targeting the 2058 mutants MG (FAM dye), the 2059 mutants MG (YY dye) and the total MG (Mg219 gene, ROX dye). One multiplex (M + IC) also included P/P targeting the UIC that was added to each PCR well (Cy5 dye). The other multiplex (M–IC) did not include these P/P and UIC was not added in these PCR wells. The multiplexes were tested against plasmid target DNA, isolated (to test the amplification of one target, as would occur in most clinical samples) or together (to test the amplification of all of the targets together, as would occur in a kit positive control).

Preliminary experiments were performed in the presence of UIC to determine the number of copies of target DNA per well required to reach a Ct of 30 ± 3.3 for each tested target, with this range of Ct corresponding to an average signal consistent with good quality PCR curves. These amounts of DNA were then tested with both multiplexes (with or without UIC, *n* = 6) and the ΔCt were calculated. All the compared conditions provided a ΔCt ≤ 1, validating the use of internal controls in the multiplex.

### Specificity vs non-MG species

The specificity of the M + IC multiplex (2058 mutants MG (FAM dye), 2059 mutants MG (YY dye), total MG (Mg219 gene, ROX dye) and IC (Cy5 dye)) for MG DNA was tested against commercial genomic DNA of the following bacterial, yeast and viral species: *Chlamydia trachomatis*, *Chlamydophila pneumoniae*, *Clostridium difficile*, *Escherichia coli*, *Gardnerella vaginalis*, *Lactobacillus acidophilus*, *Lactobacillus brevis*, *Lactobacillus jensenii*, *Lactobacillus reuteri*, *Mycoplasma arginini*, *Mycoplasma pneumoniae*, *Mycoplasma hominis*, *Mycoplasma orale*, *Mycoplasma salivarium*, *Neisseria gonorrheae*, *Neisseria meningitidis serogroup A*, *Neisseria meningitidis Serogroup B*, *Neisseria meningitidis FAM18 serogroup C*, *Neisseria meningitidis M1883 serogroup C*, *Trichomonas vaginalis*, *Ureaplasma parvum*, *Ureaplasma urealyticum*, *Candida albicans*, *Candida glabrata*, *Herpesvirus 1 (HSV-1)*, *Herpesvirus 2 (HSV-2)* and *Herpesvirus 5 (CMV)*. These species were selected based either on their probability of being present in clinical samples tested for MG (AZM-resistant or not) or on their phylogenetic proximity to MG. All wells showed a positive signal for the UIC, validating the PCR results. No signal for any target was obtained with any of these non-MG species, while the positive control was positive for each target.

### Limit of detection (LoD)

The LoD at 95% (lowest number of DNA targets present in the PCR well required to obtain detection 95% of the time) was determined for the M + IC multiplex against Mg219, A2058C, A2058G, A2058T, A2059G and A2059C using target plasmid DNA at 5000, 500, 50, 5 and 0.5 copies/well. As no cultures of mutant MG were available, we could not spike matrices of interest (urine and ESwab) with living MG cells then extract the total RNA/DNA and perform the RT-PCR. Instead, we extracted total RNA/DNA from these matrices previously spiked with EIC and spiked the extract with target MG plasmid DNA before performing the RT-PCR. As a reference, we also performed the same RT-PCR directly on plasmid DNA in Tris-EDTA pH 8 (Ambion, Belgium) spiked with UIC. The obtained results (see Table 2) show LoD close to 5 copies/PCR well in most cases, with a LoD below 50 copies/PCR well in all cases. All wells showed a positive signal for the internal control (EIC or UIC), validating the RT-PCR results. The sensitivity boost provided by the additional rRNA targeting through the reverse transcription step was tested on total RNA/DNA extracts from MG cells. Experiments performed on this material with or without the reverse transcription step showed a 2–3-fold difference in target concentration (ΔCt of 0.83 for WT targeting in singleplex on RNA/DNA total extract from WT MG cells). This is much lower than what was anticipated as ribosomes are expected to be present at 100–10,000 per cell, and could be explained by partial RNA degradation during handling of cells and extracts.

**Table 2:** Limit of detection for each of the plasmidic DNA targets (Mg219, A2058C, A2058G, A2058T, A2059G, A2059C), with internal control (EIC for urine and ESwab and UIC for Tris-EDTA). N = 6

Target	Urine / EIC (copies/PCR)	ESwab / EIC (copies/PCR)	Tris-EDTA / UIC (copies/PCR)
Mg219	7.30	7.30	6.45
A2058C	23.40	6.45	6.00
A2058G	6.00	6.00	23.40
A2058T	49.85	6.00	6.00
A2059G	36.10	6.45	6.45
A2059C	5.65	6.00	6.00

### Co-infection interference testing

In multiplex PCR assays, competition for reagents can result in non-detection of a low-concentration target when another target, the interferent, is present in high concentration. Here, co-infection interference was tested for each target (plasmid DNA for Mg219, A2058C, A2058G, A2058T, A2059G and A2059C) using the M + IC multiplex. EIC was added to urine and to ESwab matrices, then total RNA/DNA was extracted from these spiked matrices. Target DNA was added separately to these extracts at 5× the calculated LoD. Interferent DNA at 10^6^ copies/PCR well (test conditions) or extracts (controls) were added to the wells. RT-PCR results between conditions were analyzed based on Ct and percentage of detection (number of wells with target detection versus total number of wells) as described in the methods. These results are summarized in Table 3. All wells showed a positive signal for the internal control (EIC), validating the RT-PCR results.

**Table 3:** Co-infection interference testing between the plasmidic DNA targets of the M + IC multiplex

Plasmids: 10^6^ copies 5× LoD	Mg219	A2058C	A2058G	A2058T	A2059G	A2059C
Mg219	–	NI / NI	NI / I	NI / I	NI / NI	I / NI
A2058C	NI / NI	–	SD	SD	I* / I*	I* / I*
A2058G	I / NI	SD	–	SD	I* / I*	I* / I*
A2058T	NI / NI	SD	SD	–	I* / I*	I* / I*
A2059G	NI / NI	I* / I*	I* / I*	I* / I*	–	SD
A2059C	NI / NI	I* / I*	I* / I*	I* / I*	SD	–

X / X: in urine / in E-Swab; NI: non interfering; I: interfering; I*: strongly interfering (entirely cancels signals at 5× the LoD); −: same target; SD: same dye. n = 3

A slight interference (ΔCt = 0.61 while 2SDrep = 0.56) was noted in urine for A2058G targeting when Mg219 is the interferent. Strong interference was found between mutants, entirely canceling signals for the targets at 5× the LoD, which was expected since they share the same primers. Also, interferences were obtained for MG219 targeting when the interferents are A2058G (ΔCt = 2.32) or A2058T (ΔCt = 2.56) in E-Swab and A2059C (ΔCt = 1.38) in urine. This is not a problem because the MG219 target could always be detected.

### Endogenous substance interference testing

Substances potentially present in tested clinical samples can have the ability to interfere with PCR assays. In our case, the protocol for testing of endogenous substance interferences with the M + IC multiplex was based on the protocol performed to validate the S-DiaMGTV™ kit from Diagenode. Tested substances and matrices were as follows: blood 1% in urine, albumin 1 mg/ml in urine and blood 10% in ESwab. Tested substances were added to matrices spiked with EIC as described in the methods. Controls consisted in similar matrices without addition of tested substances. Total RNA/DNA was extracted and the extracts were spiked with plasmidic DNA targets (Mg219, A2058C, A2058G, A2058T, A2059G and A2059C) at 5× the calculated LoD before performing the RT-PCR. Conditions with and without endogenous substances were compared and analyzed as described in the methods. These results (*n* = 12) showed no interference of the tested substances in any tested condition. All wells showed a positive signal for the internal control (EIC), validating the RT-PCR results.

### Testing of clinical samples

**Fig. 1:**
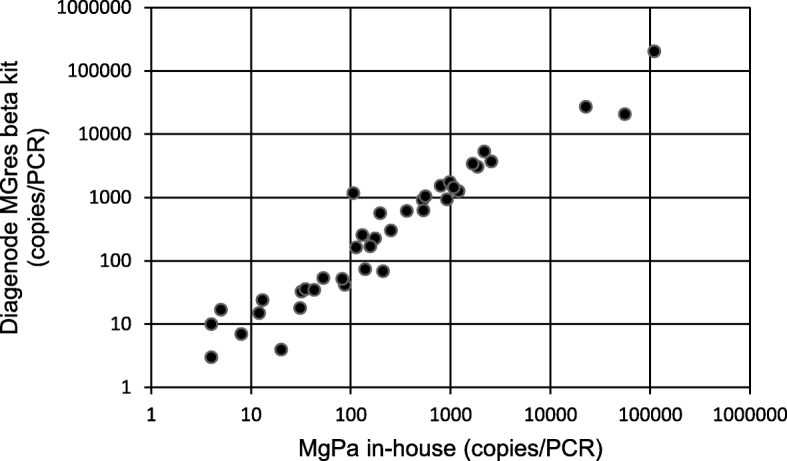
Quantitative comparison between the M + IC multiplex (Diagenode MGres beta kit) and the Statens Serum Institut reference assay (MgPa in-house)

43 clinical samples were selected based on prior routine diagnostic pyrosequencing assay, including both azithromycin sensitive MG and mutant types accounting for the majority of azithromycin resistance mutations seen in MG clinical samples. Distribution of sample types according to resistance type is shown in Table 4. When the M + IC multiplex was applied to these 43 clinical samples, results showed a 95% fit between the M + IC multiplex and the reference assay (Table 4). Negative results (no target detection) in both the reference and M + IC assays were interpreted as RNA/DNA degradation during storage of the clinical samples. Detected discrepancies between M + IC multiplex results and Statens Serum Institut records revealed a mutant that was initially erroneously typed, as well as a possible mixed infection not identified in the reference test (2058 mutant + 2059 mutant in the same sample: see [b] in Table 4). After resolving the discrepancies, the M + IC results were 100% concordant with the reference assay. Quantitative results (Fig. 1) produced for both PCR assays showed correlated data between both assays (r^2^ = 0.866).

**Table 4:** Description of samples used in the pre-validation of the M + IC multiplex (Diagenode MGres beta kit)

Mutation *E. coli* numbering	Comment	Number of samples included in study	Number of samples correctly identified	Sample type
WT	Wild-type	8	8	3 M urethra
				3 M urine
				1 F cervix
				1 F urine
A2059G	Common	9	9	2 M urethra
				2 M urine
				3 F cervix
				1 F urethra
				1 F urine
A2058C	Rare	4	4	4 F cervix
A2058G	Most common	8	8	3 M urethra
				2 M urine
				3 F cervix
A2059T	Very rare	0	ND	
A2058T	Relatively rare	9	8^a^	3 M urine
				4 F cervix
				2 F urine
A2059C	Rare	2	1^b^	1 M urethra
				1 F cervix

*F* female, *M* male, ^a^: one sample not *M. genitalium* positive (both assays); ^b^: one sample with dual populations (one with 2058 mutation and one with 2059 mutation); one misclassified in the sample selection step as it was an A2058C mutation (correctly identified by MGres)

## Discussion

Resistance to azithromycin in MG is caused by mutations of the 23S rRNA gene sequence at position 2058 and 2059 [14]. While the presence of azithromycin resistant MG in the urogenital tract prior to treatment leads to recurrent infections, the use of that antibiotic on sensitive MG is also able to trigger such mutation-induced resistance. Recent studies have shown that the prevalence of resistance to macrolide treatment is rising in different parts of the world [21–25], although this is not observed in every study [5], and it can vary according to the studied population [26]. In 2017, the European guideline for the management of pelvic inflammatory disease changed the first-line treatment from azithromycin to moxifloxacin in cases of confirmed MG infection [27]. Tucker and Ong [28] emphasized the usefulness of pertinent MG diagnostic kits. In addition, undiagnosed MG co-infections with other bacteria such as *Chlamydia trachomatis* or *Neisseria gonorrhoeae* can expose MG to suboptimal azithromycin doses, potentially leading to macrolide resistance [6]. Availability of sensitive and specific NAATs targeting MG and its azithromycin-resistance mutations is therefore essential. The four-color multiplex real-time PCR assay prototype tested in this article has been devised to answer that need.

The aim of the prototype was to detect both MG and 5 azithromycin-resistance mutations in the same assay. This assay being purely based on the amplification of target nucleic acids, it requires only classical primers and fluorescent dye-coupled probes and can be performed using classical real-time PCR cyclers in any routine clinical lab, with no need for additional expensive equipment or software. Therefore, the entire set of data is obtained in a single PCR process, without the need for additional testing such as Sanger sequencing to detect mutations. This strategy is both time- and cost-efficient. The specificity of the prototype for its targets has been investigated first against both plasmid DNA and MG genomic DNA. After validation of the detection of each of the targets, potential unwanted cross-reactions were tested between targets and against genomic material from 27 other pathogenic and commensal species that are either potentially present in the sampling sites or that are phylogenetically close to MG. No cross-reaction was detected, allowing us then to consider the structure of the prototype we wanted to build. It was decided for this prototype to use different dyes for 2058 and 2059 mutations, allowing future comparison with pyrosequencing during the clinical validation. This also enables detection of mixed 2058- and 2059-mutated populations in a sample. The addition of an internal control, a routine step for all IVD kits from Diagenode s.a., to the prototype was also tested and showed no deleterious consequences for the detection of the targets.

The limit of detection is a critical factor to consider when targeting pathogens such as MG, as undetected small amounts of azithromycin-resistant MG present in co-infections or remaining after treatment can participate in increasing the propagation of this pathogen in the population and cause recurrent infections in the patients. Azithromycin-resistance mutations are located on a gene coding for rRNA, it was therefore decided to use a real-time reverse transcription PCR assay instead of a real-time PCR assay. This resulted in an increase in the number of potential targets in clinical samples by targeting both the gene coding for the 23S rRNA and the 23S rRNA itself, increasing the sensitivity by a 2–3-fold factor. For each target in each tested matrix (urine, ESwab and Tris-EDTA), we aimed for an optimal LoD of 5 copies/PCR, with the highest acceptable LoD of 50 copies/PCR. This objective was reached, with most LoD being close to 5 copies/PCR.

Interferences are other critical factors for a NAAT, and testing of these is a required step when performing the analytical validation of such an assay. First, if one target is highly concentrated, another target present at low concentration can provide a negative result (co-infection interference). When tested with our prototype, only non-problematic interferences were observed between targets with different dyes. Targets sharing the same dye could obviously not be investigated. A second type of interference studied with our prototype was endogenous substance interference; testing the impact of substances known to be potentially present in the sample, such as blood and albumin in our case, on target detection. No such interferences were observed here.

Analytical validation could only be performed on plasmid DNA and on genomic DNA extracted from cultures. Validation of the prototype on real clinical samples was required. A beta-kit was provided for testing at the Statens Serum Institute. When tried on a panel of 43 clinical samples, this prototype proved at least as sensitive and specific as the in-house NAAT + pyrosequencing protocol used routinely.

## Conclusions

This study reports the analytical and clinical evaluation of a new NAAT prototype targeting the presence of *Mycoplasma genitalium* and of 2058 and 2059 azithromycin-resistance mutations in that pathogen. This prototype has been developed in collaboration with the Diagenode s.a. company, which used it as a basis for the development of the commercial S-DiaMGRes IVD kit. The S-DiaMGRes kit is intended to be used as a second-line diagnostic tool targeting MG and its 2058 and 2059 macrolide-resistance mutations on MG-positive samples.

## Data Availability

The datasets generated and analyzed during the current study are not publicly available because they belong by contract to Diagenode s.a. Only datasets included in the manuscript have been made publicly available. Other datasets may be available from the corresponding author on reasonable request and only after approval from Diagenode s.a.

## Abbreviations

AZM: Azithromycin
EDTA: Ethylenediaminetetraacetic acid
EIC: Extraction & inhibition control
IC: Internal control
IVD: In vitro diagnostic
LoD: Limit of detection
MG: *Mycoplasma genitalium*
MRMM: Macrolide-resistance-mediating mutations
NAAT: Nucleic acid amplification test
NCNGU: Non-chlamydial non-gonococcal urethritis
P/P: Primers and probe
PCR: Polymerase chain reaction
rRNA: Ribosomial RNA
RT-PCR: Reverse transcriptase polymerase chain reaction
UIC: Universal inhibition control
WT: Wild type
